# Sustainability of an electronic technology-based intervention in general practice targeting improved detection and monitoring of the interrelated chronic vascular diseases

**DOI:** 10.1186/s12911-026-03572-4

**Published:** 2026-05-28

**Authors:** Julia L. Jones, Koen Simons, Jo-Anne Manski-Nankervis, Peter Shane Hamblin, Natalie G. Lumsden, Maximilian P. De Courten, Edward D. Janus, Craig L. Nelson

**Affiliations:** 1 University of Melbourne, Melbourne, Australia; 2 Western Health Chronic Disease Alliance, Melbourne, Australia; 3 Mitchell Institute for Education and Health Policy, Victoria, Australia

**Keywords:** Electronic medical records, General practice, Chronic kidney disease, Diabetes

## Abstract

**Background:**

Chronic kidney disease (CKD), type 2 diabetes (T2D) and cardiovascular disease cause substantial morbidity and mortality. Our earlier randomised stepped-wedge trial targeted these conditions using an electronic technology audit tool combined with benchmarking, education, monitoring and support to general practices. This study aimed to determine if changes seen in the original trial persisted 12 months later.

**Methods:**

The study commenced at completion of the original 80-week trial. Practices received: final training session, resource folder, ongoing electronic technology tool access; other intervention components were withdrawn. Active patients (≥3 visits within last 24 months) aged ≥ 18 years within eight practices were included. Pre-defined variables from the original trial for which the credible interval (CI) did not include one, as well as two additional variables were re-assessed 12 months later. De-identified data were analysed using R version 3.5.1 with Bayesian generalised linear mixed model with practice specific random intercept and linear slope for time. Net effect odds ratio (OR) reflects the combined outcome of the original trial and subsequent follow-up period.

**Results:**

37,813 patients were included at study end. Net OR and 95% CI showed: increased CKD diagnostic testing in those at risk (OR 1.4, CI 1.2–1.6), increased coded CKD diagnosis (OR 1.9, CI 1.6–2.2) and increased uACR testing in patients with T2D (OR 1.9, CI 1.4–2.5). When considering the proportion of patients with CKD on recommended management among all active patients aged ≥ 18 years, there were also increased patients with CKD prescribed ACEI/ARBs (OR 1.8, CI 1.5–2.3) and prescribed statins (OR 1.8, CI 1.4–2.2). There was no sustained increase in T2D diagnostic testing in those at risk.

**Conclusions:**

Improvements strengthened in five areas including three out of four pre-defined variables that improved in the original trial and two additional variables that also showed improvement in the original trial, suggesting lasting benefits with ongoing electronic technology tool access. Further investigation incorporating control practices and qualitative research investigating which components best promote long-term changes would be beneficial.

**Registration number:**

ACTRN12617000335392. Date registered: 3 March 2017.

**Supplementary Information:**

The online version contains supplementary material available at 10.1186/s12911-026-03572-4.

## Background

Chronic kidney disease (CKD), diabetes and cardiovascular disease (CVD) are interrelated chronic vascular diseases that are common [1], share multiple risk factors [2] and have high morbidity and mortality [3, 4] leading to substantial healthcare expenditure [5–7].

Multiple randomised controlled studies have shown the potential for electronic technology tool-based interventions to improve the detection and management of these conditions [8–28], but there are few studies assessing electronic-technology tool-based interventions targeting these three interrelated chronic vascular diseases together [19, 29]. There are also few data on the sustainability of electronic technology tool-based interventions with only one of these studies having longer than 2 years follow-up, finding increased guideline-recommended pathology testing in patients with diabetes [15].

Our randomised stepped wedge trial [30], Chronic Disease IMPACT (Chronic Disease early detection and Improved Management in PrimAry Care ProjecT), assessed an electronic technology tool-based intervention (also comprising education to general practices, assistance with quality improvement audit planning, benchmarking, monitoring and support) targeting the interrelated chronic vascular diseases together in a general practice setting. In the first 16-week period, none of the practices received the intervention and the longest length of follow-up from intervention commencement (when the first practices in this stepped wedge trial received the intervention) was 64 weeks. This original Chronic Disease IMPACT randomised stepped-wedge trial (henceforth referred to as the original trial) consisted of a convenience sample of practices, rather than randomly selected practices because of the resources available for this study.

Sample size calculation was not conducted prior to commencing this original trial due to the limited availability of sample size calculation tools for stepped wedge trials prior to commencement in 2016, which risks introducing type II error. A post-hoc power calculation was included in the supplementary materials of the original trial, this did not include correction for multiple comparisons, given that such post-hoc calculations are of little utility in the interpretation of results. This is a limitation of the original study, and in future studies it would be beneficial to conduct power calculations including correction for multiple comparisons where relevant. Generally, it will not be possible to increase the sample size for the sustainability follow-up study, but power could be influenced by selecting the number of extended follow-up measurements - which was limited to one - and by specifying an appropriate non-inferiority limit. Foregoing a priori power calculation, this study has focussed on estimating the effect sizes and interpreting the joint sustainability over multiple target outcomes.

The original trial found increased diagnostic testing for CKD in those at risk, increased coded diagnosis of CKD, increased diagnostic testing for type 2 diabetes (T2D) in those at risk and increased urinary albumin:creatinine ratio (uACR) monitoring in patients with diagnosed T2D. It also found reduced eye examinations coded in the EMR in patients with diagnosed T2D. Because practices were able to choose from many areas including risk factor detection, diagnosis and disease monitoring/management for CKD, T2D and CVD, there were many variables assessed in the original trial, 45 in total, with many variables dependent upon other variables.

In the original trial when considering just the population with a diagnosis of CKD as a denominator, the proportions of patients with CKD prescribed an angiotensin-converting enzyme inhibitors (ACEI) or angiotensin 2 receptor blocker (ARB) and those prescribed a statin did not increase. However, when considering all active patients aged ≥ 18 years as a denominator and assessing the proportions of patients within this larger population with a diagnosis of CKD prescribed an ACEI/ARB or prescribed a statin, this proportion did increase. This relates to the increase in patients being diagnosed with CKD associated with the intervention shifting the denominator of the group being assessed. By assessing ACEI/ARB and statin prescriptions just amongst those with a diagnosis of CKD, the effect was masked because of the concomitant increase in CKD diagnosis. Therefore, although the introduction of non-pre-defined variables increases the risk of over-stating the effectiveness of the intervention, it was felt that it was important to assess the sustainability of changes to ACEI/ARB and statin prescription using the whole population as a denominator. We decided to assess ACEI/ARB and statin prescription in CKD with both denominators for transparency.

All elements of the original intervention, apart from the electronic technology tool itself, depended on trial staff for their provision. This study aimed to assess the sustainability of the Chronic Disease IMPACT intervention by assessing whether those changes seen at the completion of the original trial would continue to improve in the 12 months after completion of the original trial. Specifically, if additional disease testing and diagnosis would continue during the follow-up period, in the presence of ongoing access to the electronic technology tool, without ongoing trial staff support.

## Materials and methods

Detailed methods of the original trial have been described elsewhere [30]. The intervention involved: an electronic technology tool that extracted data from general practice electronic medical records (GP EMR) and generated graphs and lists for audit; disease specific and electronic technology tool education to general practice staff; assistance with quality improvement audit plan development, benchmarking, monitoring and support. Education sessions were conducted by specialist physicians. There was additional education provided by project officers who also assisted practices with the other intervention components. The intervention was assessed with a randomised stepped wedge trial in which nine general practices (each considered a cluster) were randomised to gain access to the intervention in different periods. Each successive period was of 16 weeks duration.

This study (henceforth referred to as the sustainability study) provides an extended follow-up to the original trial. After five periods (by which point all the practices had received the intervention for at least one 16-week period), the 12-month sustainability period commenced. At the start of the sustainability study, all the practices received a final training session on quality improvement and electronic technology tool use as well as a folder containing relevant resources. During the sustainability study, practices maintained access to the electronic technology tool, but did not receive any of the other intervention components (education, assistance with quality improvement audit plan development, benchmarking, monitoring and support).

General practices were eligible for inclusion in the original trial based on the following requirements: Located in Victoria, Australia; >2000 patients attending the practice; licence for (or willing to install) Pen Computer Systems Clinical Audit Tool (Pen CAT); no participation in other quality improvement projects with similar targets; practice using one of three EMR systems (Medical Director, Best Practice or ZedMed) continuously for ≥two years. Those practices that participated in the original trial were eligible to participate in the sustainability study and all practices from the original trial were willing to continue in this subsequent study. Active patients (having attended their practice ≥ 3 times within the last 24 months) aged ≥ 18, were included as participants in each period.

De-identified patient data were extracted from general practice EMRs using the Pen Computer Systems data extraction tool. Variables with evidence for effect at the end of the original trial (the end of period 5) [30] were re-assessed 12 months later at the end of the sustainability study to test sustainability of outcomes. These variables included: testing for kidney disease in those at risk; coded CKD diagnosis; testing for diabetes in those at risk; urine albumin:creatinine ratio testing in people with diagnosed T2DM; patients with CKD prescribed ACEI/ARB; patients with CKD prescribed statins; up-to-date coded eye checks in people with diagnosed T2DM. There was not one cohort of patients followed through the entire study, but a cross-section of all active patients ≥ 18 years of age assessed at the end of each 16-week period of the original trial and at the end of the 12-month sustainability study.

Analysis was conducted with R version 3.5.1. Data were analysed using a Bayesian generalised linear mixed model with a practice specific random intercept and a linear slope for time. In addition, we included a term for decline or increase in effect after the end of the 12-month follow-up period. As there were no control practices during the sustainability period, a linear trend for time was assumed instead of allowing an unstructured effect of time, whereas the original trial analysis [30] was conducted considering time as a random intercept. Linear extrapolation addresses the risk of confounding secondary to the absence of a control group and reduces but does not eliminate this risk. It explicitly assumes that independent changes in the target outcomes over time are gradual and linear over the whole study period, including both the original trial time and the extended follow-up time. Data completeness was assessed by monitoring for unexpected decreases in patient numbers or in variables such as completion of pathology tests, that would not be detected if pathology test codes changed without study staff awareness. In the original trial, one practice was excluded because of a practice merge affecting data quality, leaving eight practices in the analysis and this practice was also excluded in the sustainability study, although a sensitivity analysis including this practice was also conducted.

The original trial main effect OR was defined as the effect seen with practices receiving the full intervention as per the randomisation schedule in the initial five 16-week periods of the original stepped wedge trial.

The change in effect OR was defined as the change from the effect size seen in these five initial periods of the original trial in which practices received the full intervention, to the effect size observed in the 12-months of the sustainability study. An odds ratio of one indicates the effect size of the original trial was maintained during the sustainability study and if greater than one indicates an additional improvement in performance during the sustainability study beyond what was seen in the original trial.

The net effect OR was defined as the product of the original trial main effect OR and the change in effect OR. This reflects the combined outcome of the original trial and sustainability study. Outcomes were considered sustainable if the 95% CI for net effect OR did not include one, meaning a change seen with the combined effect of the original trial and the pared-back intervention of the sustainability study. Being a Bayesian analysis, the emphasis is on estimation, with the 95% CI representing a 95% belief that the parameter is within the calculated interval. Analysis adjusting for multiple comparisons was conducted using Dunn–Šidák to account for the 45 outcomes analysed in the original trial.

This research was approved by the Western Health Low Risk Human Research Ethics Panel, HREC/16/WH/124. Each practice provided consent to participate, however there was a waiver of consent for patients within the practices given that only their de-identified data would be analysed and only aggregated data published. It was registered with the Australian New Zealand Clinical Trials Registry: ACTRN12617000335392.

## Results

Practice enrolment commenced in November 2016. Clinical data were first collected in April 2017, data at the end of period 5 were collected in July 2018 and data at the end of the sustainability study were collected in August 2019. This took place according to schedule with the trial ending at the planned time.

**Fig. 1:**
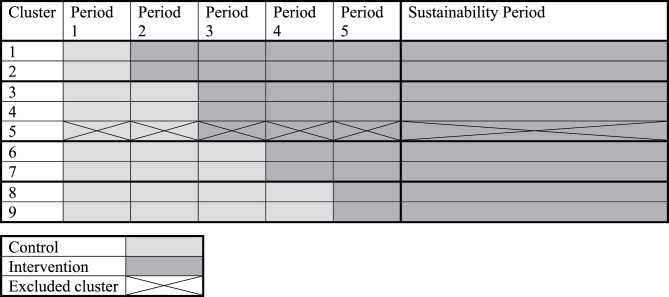
Overview of stepped wedge trial with additional sustainability period (showing excluded practice)

**Fig. 2:**
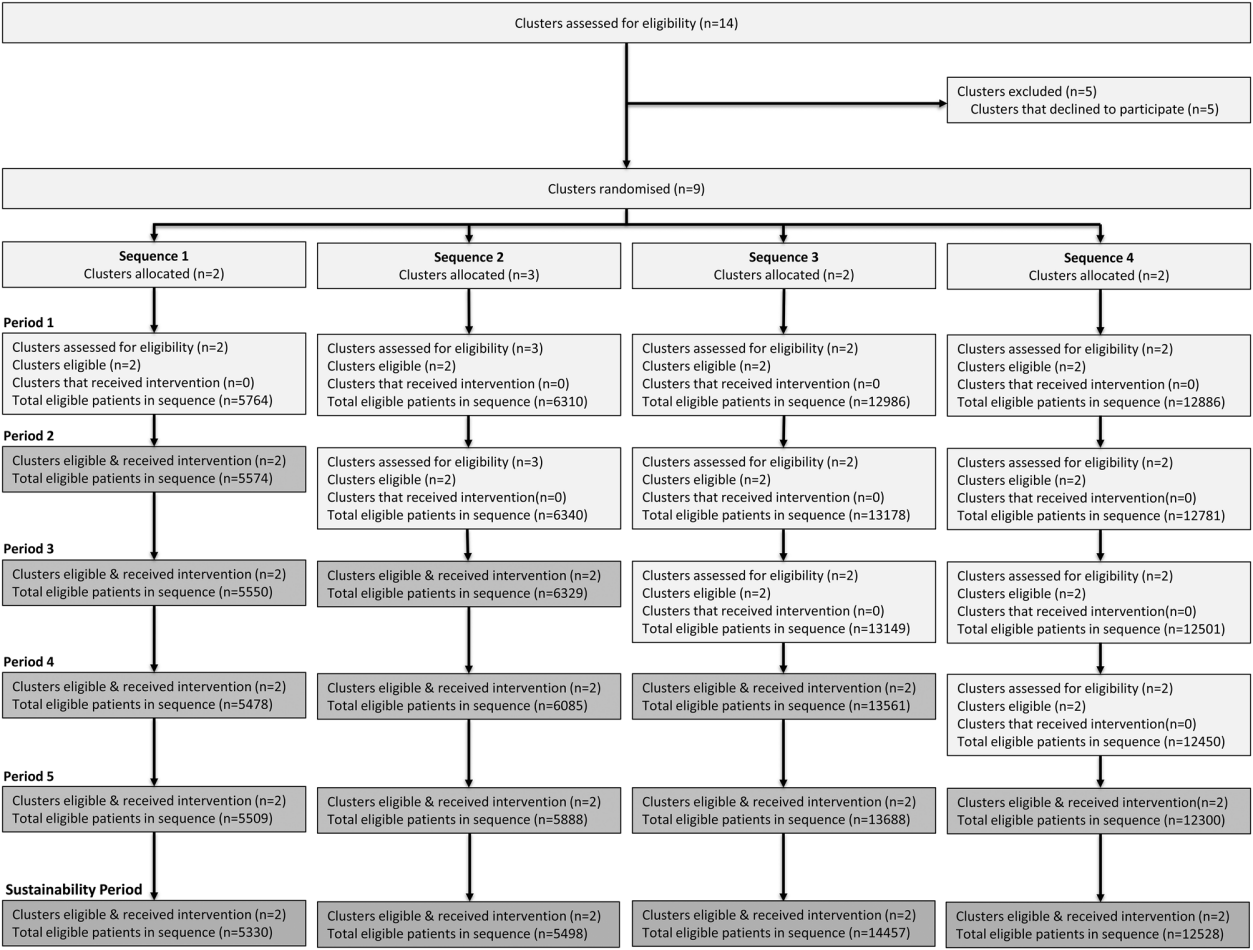
Participant flow diagram

Only eight practices were included (as with the original trial analysis) due to compromised data quality in one practice resulting from a recent merging of practices with different electronic medical record systems (see Fig. 1). A sensitivity analysis was conducted which included the practice that was excluded from the main analysis. Those variables that had net effect OR with CIs not including one in the main analysis also did not include one in the sensitivity analysis (see Supplementary Materials Table 1). Figure 2 summarises the numbers of practices and patients in sequences and periods. Henceforth, only the eight included practices are discussed.

Practices were located 12 to 165 kilometres from the centre of Melbourne, Victoria, Australia. One was situated in an inner metropolitan area, four outer metropolitan areas and three within inner regional areas. Six practices had private and two had corporate ownership. Two practices bulk-billed exclusively (where there is no additional charge to patients on top of the government rebate provided to patients to fund their care). The other practices required some patients to pay fees in addition to the government rebate. All practices were in geographical areas in the lowest four quintiles with respect to index of relative socio-economic disadvantage scores [31].

Aggregated baseline data prior to any of the practices receiving the intervention were collected in April 2017 [32] and results from the stepped wedge trial analysing data from April 2017 until July 2018 are detailed elsewhere [30].

Only data coded within the EMR was extracted with the data extraction tool. Therefore, if a doctor had documented diagnoses, observations, examinations, investigations or other relevant data using free text in a progress note, this was not captured. Additionally, pathology testing was only detected if electronically transferred from the pathology company to the EMR; scanned paper records were not included. It was not within the scope of this study to investigate the number of diagnoses, examinations and pathology tests that were not able to be detected by the data extraction software.

In April 2017, prior to any intervention, there were 37,946 active patients aged 18 years or older in the eight included practices with median age 48 years. Over a third of patients (40%) were male. The number of active patients, age and sex distribution remained similar at the end of the original trial in July 2018 and at the end of the sustainability study in August 2019 (see Table 1).

**Table 1:** Patient demographics prior to intervention, at end of original trial and at end of sustainability study

	April 2017 (prior to any intervention)	July 2018 (end of original trial)	August 2019 (end of sustainability study)
Total active patients	37,946	37,385	37,813
Mean age (years)	48	48	48
Percentage male	40	40	41

The original trial main effect OR, the change in effect OR and the net effect OR is provided in Table 2.

**Table 2:** Summary of effects seen in original trial, change with sustainability study and net effect

	Number/denominator (proportion) of patients at baseline, in April 2017	Original trial main effect OR (CI)	Change in effect at end of sustainability study OR (CI)	Net effect (original trial and sustainability study combined) OR (CI)
Diagnostic testing for CKD in those at risk (*n* = at risk of CKD)	3,630 / 18,698 (19%)	1.3 (1.2–1.3)	1.1 (1.0–1.2)	1.4 (1.2–1.6)
Coded CKD diagnosis n = all active patients ≥ 18 years)	1,709 / 37,946 (4.5%)	1.3 (1.2–1.4)	1.4 (1.3–1.6)	1.9 (1.6–2.2)
Up-to-date uACR testing in patients with T2D (*n* = T2D diagnosed)	1,508 /2,715 (56%)	1.6 (1.4–1.8)	1.2 (0.97–1.5)	1.9 (1.4–2.5)
Diagnostic testing for T2D in those at risk (*n* = at risk of T2D)	4,143 / 12,180 (34%)	1.2 (1.1–1.3)	0.89 (0.82–0.98)	1.1 (0.95–1.2)
Up-to-date coded eye examination in T2D (*n* = T2D diagnosed)	1,046 / 2,715 (39%)	0.86 (0.75–0.98)	1.1 (0.91–1.4)	0.96 (0.71–1.3)
Patients diagnosed with CKD and prescribed an ACEI/ARB (*n* = CKD diagnosed)	1,105 / 1,709 (65%)	0.97 (0.84–1.1)	0.89 (0.71–1.1)	0.86 (0.62–1.2)
Patients diagnosed with CKD and prescribed an ACEI/ARB (*n* = all active patients ≥ 18 years)	1,105 / 37,946 (2.9%)	1.3 (1.2–1.4)	1.4 (1.2–1.6)	1.8 (1.5–2.3)
Patients diagnosed with CKD and prescribed a statin (*n* = CKD diagnosed)	953 / 1,709 (56%)	1 (0.87–1.1)	0.91 (0.74–1.1)	0.9 (0.71–1.3)
Patients diagnosed with CKD and prescribed a statin (*n* = all active patients ≥ 18 years)	953 / 37,946 (2.5%)	1.3 (1.2–1.4)	1.4 (1.2–1.6)	1.8 (1.4–2.2)

CKD: chronic kidney disease

T2D: type 2 diabetes

uACR: urine albumin:creatinine ratio

ACEI: angiotensin-converting enzyme inhibitor

ARB: angiotensin 2 receptor blocker

**Fig. 3:**
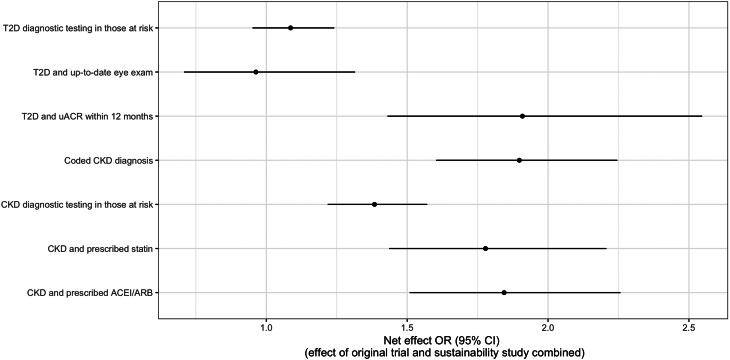
Sustainability of variables from the original trial. CKD: chronic kidney disease, T2D: type 2 diabetes, uACR: urine albumin:creatinine ratio, ACEI: angiotensin-converting enzyme inhibitor, ARB: angiotensin 2 receptor blocker

Analysis using the net effect OR is shown in Fig. 3 and reveals a sustained improvement in five areas; three out of four pre-defined variables and two additional variables that showed an improvement in the original trial. These variables with sustained improvement consist of diagnostic testing for CKD in those at risk with an odds ratio (OR) of 1.4 and a 95% CI of 1.2–1.6; coded CKD diagnosis (OR 1.9, CI 1.6–2.2), patients with diagnosed CKD prescribed an ACEI/ARB (OR 1.8, CI 1.5–2.3), patients with diagnosed CKD prescribed a statin (OR 1.8, CI 1.4–2.2) and uACR testing in patients with diagnosed T2D (OR 1.9, CI 1.4–2.6). There was no sustained increase in diagnostic testing for T2D in those at risk (OR 1.1, CI 0.95–1.2) and no sustained reduction in up-to-date coded eye examinations in T2D (OR 0.96, CI 0.71–1.3). In order to account for multiple comparisons, for each outcome and its CIs, a revised alpha has been calculated using the Dunn-Sidàk correction method with a multiplicity factor of 45 (the number of outcomes in the original trial). This revised alpha of 0.00139 has been used to calculate 99.886% CIs (see Supplementary Materials Table 2). Although the revised CIs were wider, there was no difference to plausible outcomes compared to the main analysis.

## Discussion

This research has shown that an electronic technology tool-based intervention in general practice to facilitate clinical audit, with additional components including education, benchmarking and assistance with audit planning, monitoring and support, can have sustained effects and further enhancements to performance 12 months later when practices have ongoing access to the electronic technology tool without further access to the additional supporting intervention components. It showed sustained improvement in five variables; in three out of four of the variables showing improvement in the original trial and in two additional variables that showed improvement in the original trial. These results were found for increased CKD diagnostic testing in those at risk, increased coded diagnosis of CKD, increased proportion of patients with diagnosed CKD prescribed an ACEI/ARB, increased proportion of patients with diagnosed CKD prescribed a statin and increased uACR testing in patients with diagnosed T2D. Sustainability in the majority of areas that showed initial improvements reinforces the findings of the original trial.

The implications of these sustained improvements have substantial clinical importance. The sustained increase in diagnostic testing for CKD in those at risk has the potential to improve disease detection, providing opportunities to commence timely management strategies. Patients who have uncoded diagnoses have been shown to be less likely to receive treatment according to guideline recommendations [33]. The sustained increase in coded diagnoses of CKD may facilitate adherence to guideline recommended treatment if decision support software is used. Currently, a lot of available decision support software options require a coded diagnosis to identify target patients, however this is likely to change into the future as natural language processing is becoming more sophisticated and decision support software will be increasingly likely to search through free text to identify target patients. The sustained increase in uACR testing in people with T2D offers patients an opportunity for earlier diagnosis of CKD. When CKD is diagnosed in a timely manner, patients with or at risk of progressive CKD can be referred to nephrologists which has been shown to slow disease progression and improve survival [34].

This study showed a sustained increase in the proportion of patients with diagnosed CKD prescribed recommended medication (ACEI/ARBs and statins) when considering all active patients ≥ 18 years as the denominator. It did not show an increase in the proportion of patients with diagnosed CKD prescribed these recommendations when just considering those patients diagnosed with CKD. This phenomenon may relate to the increased diagnosis of CKD associated with the intervention. The overall increased proportion of patients with CKD on ACEI/ARBs and statins is of great clinical relevance given that in CKD ACEI and ARB have been shown to reduce cardiovascular events, progression of kidney disease [35] and mortality [36] and statins have been shown to reduce cardiovascular events as well as mortality [37].

There was an increase in diagnostic testing for T2D in those at risk in the original study. There is evidence that this improvement was not fully sustained, however given that the upper limit of the change in effect OR was very close to one (at 0.98), it is possible that there was actually minimal reduction in efficiency in the sustainability study. The moderate improvement seen in the original trial could potentially have been due to a type I error. Alternatively, a reduction in effect seen in the sustainability study may reflect practices shifting the focus their quality improvement efforts over time. It would be beneficial to assess this further in subsequent studies.

The original trial showed a reduction in coded eye examinations at the recommended frequency in patients with T2D. For coded eye examinations to be captured, clinicians were required to enter this data into the EMR in a specific manner and it is likely that many eye examinations were performed but not formally documented in this way. The sustainability study showed no evidence of a sustained reduction in coded eye examinations, which is reassuring given that the coding of this data can make it easier for clinicians to ensure that such monitoring is up-to-date.

Longer term follow-up of quality improvement initiatives is of great clinical relevance but is frequently not assessed in research, potentially due to the ongoing costs of providing such programs. One cluster randomised study from the United States assessing decision support for diabetes management in primary care with longer term follow-up (patients observed for a mean of 32 months), like Chronic Disease IMPACT found improved urine albumin testing [15]. It also found improvements to some other disease monitoring measures in the intervention group [15]. This US study provided the full intervention for the duration of the study [15], rather than an initial period with the full intervention followed by a pared back intervention like in our study. Some components of the Chronic Disease IMPACT intervention have substantial costs, particularly labour costs associated with the education, assistance, monitoring and support. Access to the electronic technology tool does not require these labour costs, so if practices refine their clinical auditing process during the initial full intervention and are then able to maintain their initial gains with only ongoing access to the electronic-technology tool, this offers an affordable option for ongoing quality improvement and enables the scalability of such programs.

Strengths of this study include its length of follow-up, which is longer than most other studies assessing electronic technology-based quality improvement tools targeting chronic disease in general practice. This study included both metropolitan and non-metropolitan practices was set in the ‘real world’ with practices attending to their usual workload. A large number of patients were included in the study.

Pragmatic real-world trials are challenging to conduct with inevitable limitations. In this study limitations include the risk of selection bias associated with a convenience sample, which was used because a random selection of practices was not feasible given available resources. Participating practices may have been more enthusiastic participants in clinical audit, which may have affected their performance. Study design provided no control practices in the sustainability period, and the possibility of another cause for the sustained improvements seen in this study is not controlled via the design, nor fully controlled in the statistical analysis. Linear extrapolation of temporal trends was used, which controls for longer term time trends, but not for a sudden change between the initial study and the extended follow-up. The knowledge of being monitored can affect behaviour and it is possible that some of the effect seen in the sustainability study relates to this, however practice staff reported being so busy that they had difficulty finding time to do audit plans, making it less likely that much time was spent thinking about being monitored and less likely to impact performance. The original trial included 45 variables. Assessing large numbers of independent variables increases the risk of type I error, and although this increase would be less severe in this study, since the variables were not all independent, it would still be inflated compared to assessing a primary outcome. In retrospect, the use of a composite primary outcome in the original trial would have addressed this issue and would be worth considering in other such trials assessing a broad range of areas. Additional variables were used to assess the prescription of ACEI/ARB and statin medications for patients with CKD. Non-pre-defined variables increase the risk of bias, and to avoid this issue, when designing future studies, it would be worthwhile closely considering how changes to one variable may affect another in order to ensure that an appropriate denominator for each variable is selected. We did not examine other clinical measures which did not show an improvement in the original trial but may have shown improvements subsequently.

Further research assessing electronic tool-based interventions to improve the detection and management of people with and at risk of chronic disease with a control group available during long-term follow up would be beneficial to confirm the persistence of effects with just electronic technology tool availability with no ongoing study staff support. Technological innovations continue to evolve and subsequent studies will likely look at novel technologies, such as another Australian electronic technology-based intervention that has taken inspiration from Chronic Disease IMPACT and uses both point of care and clinical audit tools that integrate with GP EMR software to improve the detection and management of chronic kidney disease in general practice [38]. Additionally, qualitative research investigating which elements of the intervention promoted long-term changes would be valuable.

## Conclusion

Electronic technology-based interventions in general practice have been shown to improve the detection and management of chronic disease, but there are limited data regarding the longer-term outcomes of such interventions. Our original trial showed that an electronic technology based-tool based intervention facilitating clinical audit in general practice which included benchmarking, education, monitoring and support was able to target multiple chronic diseases at the same time and lead to improved chronic disease testing, diagnosis and monitoring in some, but not all, conditions. This study assessed the sustainability of the intervention by assessing outcomes with a plausible effect, as assessed in the original trial, 12 months after completion of the original trial. It found sustained improvements in diagnostic testing for CKD in those at risk, coded diagnosis of CKD diagnosis, patients with diagnosed CKD prescribed an ACEI/ARB, patients with diagnosed CKD prescribed a statin and up-to-date uACR testing in people with T2D. The increase in diagnostic testing for T2D in those at risk and the reduction in coded eye examinations in people with T2D which were seen in the original trial were not maintained in this follow-up study. Many components of quality intervention programs are labour-intensive and limit program accessibility and longevity. This study showed the ability of practices to achieve ongoing improvements with only electronic technology tool access, 12 months after cessation of initial labour-intensive intervention components, thereby facilitating cost-effective lasting improvements. It also highlighted some of the challenges associated with undertaking a pragmatic trial in general practice aiming to address multiple medical conditions with long-term follow-up and provides learning opportunities for others conducting similar research. Further research to confirm these findings with control practices and qualitative investigation assessing which components promoted the most change would be of use. The sustained improvement in chronic disease testing, diagnosis and management seen in this study highlights the potential of electronic technology tool-based quality improvement interventions in general practice.

## Electronic supplementary material

Below is the link to the electronic supplementary material.

Supplementary Material 1

## Data Availability

Data are available upon reasonable request. Datasets generated and analysed in this study are not available to the public as per the agreement within the ethics committee approval, but are available upon reasonable request to the corresponding author subject to ethics approval.

## Abbreviations

ACEI: Angiotensin-converting enzyme inhibitor
ARB: Angiotensin 2 receptor blocker
Chronic Disease IMPACT: Chronic Disease early detection and Improved Management in PrimAry Care ProjecT
CI: Credible interval
CKD: Chronic kidney disease
EMR: Electronic medical record
Pen CAT: Pen Computer Systems Clinical Audit Tool
T2D: Type 2 diabetes
uACR: Urine albumin creatinine ratio
