# Supplementary material for: Sustainability of an electronic technology-based intervention in general practice targeting improved detection and monitoring of the interrelated chronic vascular diseases
Source: BMC Med Inform Decis Mak. 2026 May 28;26:282. doi: 10.1186/s12911-026-03572-4 (PMC13411737; doi:10.1186/s12911-026-03572-4)
Supplement: Supplementary file 1 — Supplementary Material 1 [file 12911_2026_3572_MOESM1_ESM.docx]

**Supplementary Materials**

Sustainability of an electronic technology-based intervention in

general practice targeting improved detection and monitoring

of the interrelated chronic vascular diseases

*Supplementary Materials Table 1 – Sensitivity analysis with practice affected by practice merge included (this practice was excluded from main analysis)*

|  | Original trial main effect OR (CI) | Change in effect at end sustainability study OR [CI] | Net effect (original trial and sustainability study combined) OR [CI] |
| --- | --- | --- | --- |
| Diagnostic testing for CKD in those at risk  (n = at risk of CKD) | 1.3 [1.2; 1.4) | 0.95 (0.88; 1) | 1.2 (1.1; 1.4) |
| Coded CKD diagnosis  n = all active patients ≥ 18 years) | 1.3 (1.2; 1.4) | 1.2 (1.1; 1.4) | 1.6 (1.3; 1.8) |
| Up-to-date uACR testing in patients with T2D  (n = T2D diagnosed) | 1.8 (1.6; 2) | 0.88 (0.74; 1.1) | 1.6 (1.2; 2.1) |
| Diagnostic testing for T2D in those at risk  (n = at risk of T2D) | 1.1 (1.1; 1.2) | 0.89 (0.82; 0.98) | 1 (0.89; 1.2) |
| Up-to-date coded eye examination in T2D  (n = T2D diagnosed) | 0.9 (0.8; 1) | 1.2 (0.86; 1.3) | 0.93 (0.7; 1.2) |
| Patients diagnosed with CKD and prescribed an ACEI/ARB  (n = all active patients ≥ 18 years) | 1.3 (1.2; 1.4) | 1.2 (1; 1.4) | 1.5 (1.2; 1.8) |
| Patients diagnosed with CKD and prescribed a statin  (n = all active patients ≥ 18 years) | 1.3 (1.2; 1.4) | 1.1 (1; 1.3) | 1.4 (1.2; 1.8) |

*Supplementary Materials Table 2 – Net effect adjusted for multiple comparisons using Dunn–Šidák with a multiplicity factor of 45*

|  | Net effect OR (95% CI);  alpha = 0.05 | Net effect OR (99.886% CI) adjusted for multiple comparisons; alpha = 0.00139 |
| --- | --- | --- |
| Diagnostic testing for CKD in those at risk  (n = at risk of CKD) | 1.4 (1.2; 1.6) | 1.4 (1.1; 1.7) |
| Coded CKD diagnosis  n = all active patients ≥ 18 years) | 1.9 (1.6; 2.2) | 1.9 (1.4; 2.5) |
| Up-to-date uACR testing in patients with T2D  (n = T2D diagnosed) | 1.9 (1.4; 2.5) | 1.9 (1.2; 3.1) |
| Diagnostic testing for T2D in those at risk  (n = at risk of T2D) | 1.1 (0.95; 1.2) | 1.1 (0.87; 1.4) |
| Up-to-date coded eye examination in T2D  (n = T2D diagnosed) | 0.96 (0.71; 1.3) | 0.96 (0.58; 1.6) |
| Patients diagnosed with CKD and prescribed an ACEI/ARB  (n = all active patients ≥ 18 years) | 1.8 (1.5; 2.3) | 1.8 (1.3; 2.6) |
| Patients diagnosed with CKD and prescribed a statin  (n = all active patients ≥ 18 years) | 1.8 (1.4; 2.2) | 1.8 (1.2; 2.5) |
